# Framework Development for Clinical Comprehensive Evaluation of Drugs–a Study Protocol Using the Delphi Method and Analytic Hierarchy Process

**DOI:** 10.3389/fphar.2022.869319

**Published:** 2022-05-19

**Authors:** Chi Zhang, Er-Li Ma, Bing-Long Liu, Bin Wu, Zhi-Chun Gu, Hou-Wen Lin

**Affiliations:** ^1^ School of Medicine, Tongji University, Shanghai, China; ^2^ Department of Pharmacy, Ren Ji Hospital, Shanghai Jiao Tong University School of Medicine, Shanghai, China; ^3^ Shanghai Anticoagulation Pharmacist Alliance, Shanghai Pharmaceutical Association, Shanghai, China; ^4^ Shanghai Pharmaceutical Association, Shanghai, China

**Keywords:** clinical comprehensive evaluation of drugs, framework, delphi method, analytic hierarchy process, protocol

## Abstract

Measuring the value of drugs to help make health-care decisions is a complex process which involves confronting trade-offs among multiple objectives. Although guidelines have been released for clinical comprehensive evaluation of drugs, refinement is required when considering a specific drug used in a specific disease. In this study, a two-level framework for clinical comprehensive evaluation of drugs will be developed. Six first-level indicators, including safety, efficacy, costs/cost-effectiveness, novelty, suitability, and accessibility will be evaluated according to the Chinese Guideline for Clinical Comprehensive Evaluation of Drugs. The second-level components involved in the framework will be first validated by the Delphi method and subsequently compared with one another to get the index weight based on the Analytic Hierarchy Process (AHP). The scoring criteria of each component in the framework will also be determined by the Delphi method and AHP. The scoring criteria of components representing therapeutic effects will involve both score of therapeutic effects and score of evidence quality. With the evidence of the drug to be evaluated, the score of each component will be obtained according to the established scoring criteria, and the overall comprehensive score value of the drug will be calculated, which will assist the evidence-based decision making.

## 1 Introduction

The World Health Organization model lists of essential medicines are medicines that satisfy the priority health care needs of the world population, which have been assessed and selected based on comparisons between various drug products considering many factors (Reidenberg, 2007). The reimbursement drug list or formulary represents a list of preferred medicines under the certain medical policies (Li et al., 2018). China’s National Reimbursement Drug List (NRDL) and the essential drug list (EDL) are important guidance lists aiming to provide basic medical coverage to the population of 1.4 billion, which are updated periodically. Drugs in NRDL and EDL are selected and determined by a core group of physicians, pharmacists, economists, and other healthcare professionals, synthetically assessing safety, efficacy, cost-effectiveness and other aspects of drugs (Tian et al., 2012; Liu et al., 2019). To ensure the objective evaluation, it is of great importance to measure the value of drugs using evidence-based method (Tian et al., 2012). Examples are the Canadian Agency for Drugs and Technologies in Health’s (CADTH) Therapeutic Review Framework (Tierney and Manns, 2008), and the Drug Effectiveness Review Project (DERP) in the United States (McDonagh et al., 2012), which contributed to the update of formularies (Tadrous et al., 2020). However, the two reviews may be jurisdiction-specific and the frameworks of drug evaluation are diverse in different organizations (Neumann and Cohen, 2015).

Measuring the value of drugs to help make health-care decisions is a complex process which involves confronting trade-offs among multiple objectives (Thokala et al., 2016). Frameworks for drug evaluation usually use different strategies for weighing various dimensions and deriving an overall score (Neumann and Cohen, 2015). However, numerous challenges exist when using the frameworks. First, many attributes can influence the value measurement of a drug, and there is no consensus on what dimensions and how many dimensions should be taken into account (Kaló et al., 2015). Moreover, value is an elusive target, but the true value of each component in the framework needs to be measured with a relative weight (Neumann and Cohen, 2015; Inotai et al., 2018). In July 2021, Chinese government released the Guideline for Clinical Comprehensive Evaluation of Drugs (GCCED) (National Health Commission of the People’s Republic of China, 2021), to provide evidence support for the improvement of national drug policy as well as the supply and rational use of drugs. This guideline suggested that evidence of six dimensions should be combined when evaluating drugs comprehensively, including safety, efficacy, costs/cost-effectiveness, novelty, suitability, and accessibility (National Health Commission of the People’s Republic of China, 2021). However, this is a universal guideline for clinical comprehensive evaluation of most drugs, which do not recommend the key components under each dimension due to the varied disease characteristics. Therefore, the secondary indicators under each dimension need to be further refined when considering a specific drug used in a specific disease. Furthermore, how to assign a score for an index also remains a challenge for quantifying the real value of a certain drug.

In this study, a two-level framework for clinical comprehensive evaluation of drugs will be developed. Refinement, including the components need to be evaluated and their corresponding weights in each level of the framework, will be set under the six dimensions pre-defined in GCCED. With the use of this framework, each drug will get a final overall score after the assessment of each component. The overall score will support the decision making for quantifying the value when a group of drugs need to be selected into NRDL and EDL as well as the rational use in clinical practice. The development of framework in this study can be a reference to the framework construction for clinical comprehensive evaluation of drugs.

## 2 Materials and Methods

### 2.1 Study Design

**FIGURE 1:**
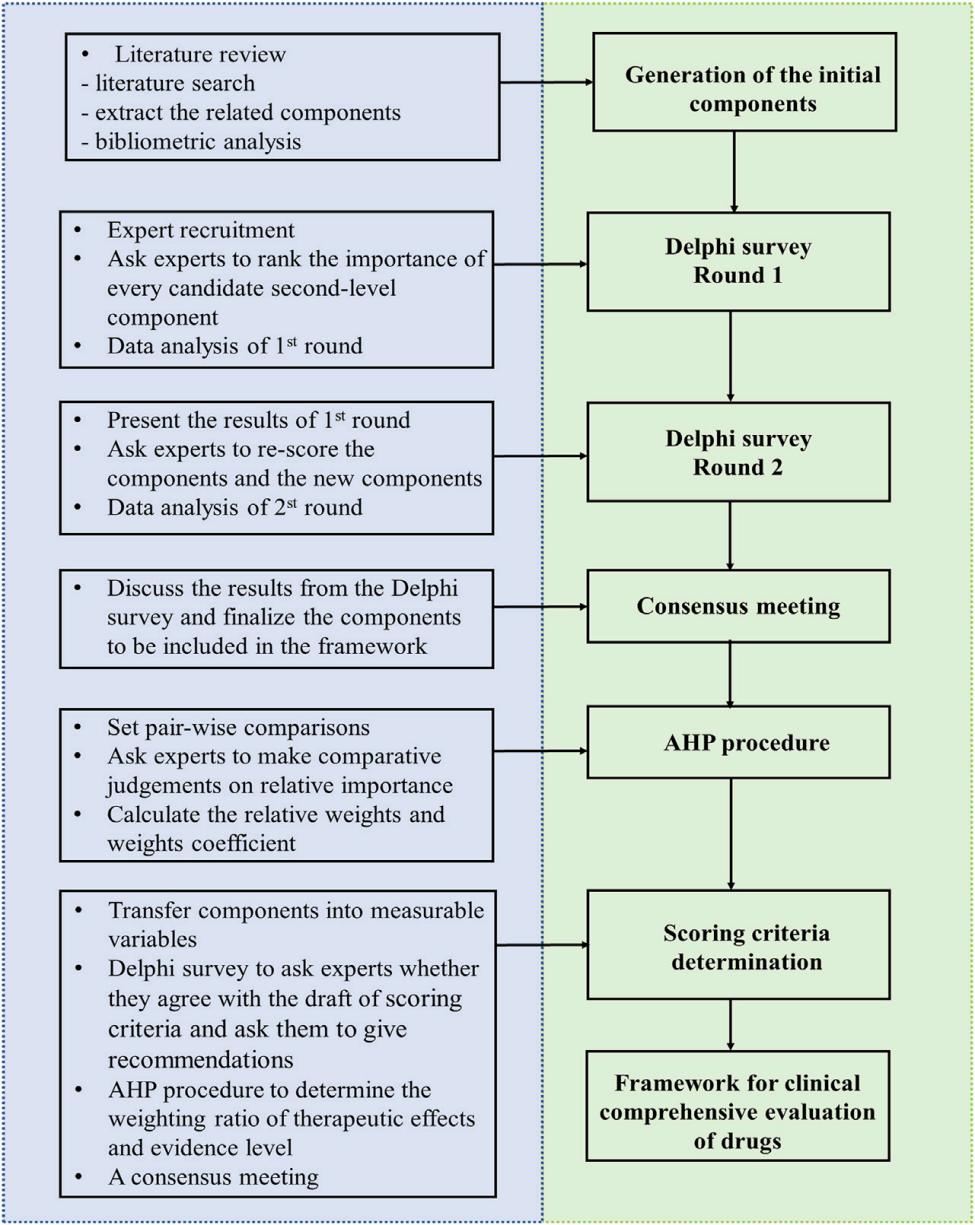
The process of developing the framework.

The objective of this study is to develop a framework to be used in the clinical comprehensive evaluation of drugs. It is descriptive-quantitative in design. The components involved in the clinical comprehensive evaluation of drugs will be firstly validated by the Delphi method and subsequently compared with one another to get the index weight based on the Analytic Hierarchy Process (AHP). The scoring criteria of each second-level component in the framework will also be determined by the Delphi method and AHP. The overview of the process is presented in Figure 1. This study started in December 2021 and is anticipated to continue until May 2023. The generation of the initial components in the framework has been completed, and the initial draft of framework has been developed. The first round of Delphi survey in ongoing.

### 2.2 Generation of the Initial Components

A two-level framework for clinical comprehensive evaluation of drugs will be constructed. Six indicators for six dimensions need to be evaluated, including safety, efficacy, costs/cost-effectiveness, novelty, suitability, and accessibility, which will be involved in the first level according to the GCCED (National Health Commission of the People’s Republic of China, 2021). The indicators at the second level need to be selected and determined using the Delphi method.

As characteristics vary from disease to disease, the components to be evaluated need to be tailored considering a specific drug used in a specific disease. For example, a framework for comprehensive evaluation of non-vitamin K antagonist oral anticoagulants (NOACs) used for stroke prevention in atrial fibrillation (AF) will be built. To identify the potential components, a scoping literature review will be firstly conducted to find candidate components reported in studies, reviews and guidelines that concern stroke prevention with NOACs in AF patients. The search will be performed in the databases of PubMed and Web of Science with the search strategies presented in Supplementary Material; Supplementary Table S1. The inclusion criteria will be: 1) concerning the use of NOACs in patients with AF; 2) guidelines of the latest edition; 3) published in English. Two reviewers will independently select the studies, reviews and guidelines, and extract the related components. Moreover, a bibliometric analysis will be conducted using the data of retrieve records including title, abstract, and keywords of each publication. The high-frequency keywords will be collected and analyzed using VOSviewer software, which can automatically subgroup closely related keywords with a default clustering algorithm (Zhong et al., 2021). Items related to efficacy and safety outcomes, pharmacoeconomics evaluation, suitability of drug use, novelty and accessibility of NOACs will be collected considering the anticoagulation therapy in AF patients. The components collected in both literature review and bibliometric analysis will be defined and preliminarily categorized into the six first-level indicators. A consultation with two reviewers and a committee of experts composing two clinical pharmacists, two cardiologists, and two pharmacoeconomists, will be convened to evaluate the structure and the components preliminarily involved in the framework. Prior to determine the draft set of the framework, the components will also be screen to meet the principles of completeness, nonredundancy, nonoverlap, and preference independence recommended by International Society for Pharmacoeconomics and Outcomes Research (ISPOR) (Marsh et al., 2016). The initial draft of framework involving two-level indicators will be used in the subsequent Delphi survey (Table 1).

**TABLE 1:** An example of initial draft of framework for clinical comprehensive evaluation of non-vitamin K antagonist oral anticoagulants in atrial fibrillation.

First-Level Indicators	Second-Level Indicators
Safety	Risk for major bleeding
	Risk for intracranial hemorrhage
	Risk for clinically relevant non-major bleeding
	Risk for life-threatening bleeding
	Other adverse reactions except bleeding
	Reversibility of overdose, life-threatening, or uncontrollable bleeding
	Whether the anticoagulant activity can be monitored
	Food-drug or drug-drug interactions
	Contraindications/use restrictions
Efficacy	Reduction of risk for stroke
	Reduction of risk for systematic embolism
	Reduction of risk for myocardial infarction
	Reduction of mortality
	Whether recommended by clinical guidelines or consensus
	Whether recommended by clinical professionals
	Clinical unsubstitutability
Costs/cost-effectiveness	Annual cost for anticoagulants
	Results of cost-effectiveness analyses
	Budget impact analyses
Novelty	Whether it is safer, more effective, or more practical than other drugs
	Whether it is a national original drug or a modified new drug
Suitability	Whether the prescriptions meet the recommendations on drug labels or clinical guidelines
	Whether it is convenient to switch to another NOAC
	Whether it is convenient to use on perioperative management
	Patients’ adherence (taking medication irregularly or stopping taking medicine by oneself)
	Dose frequency of the NOAC
	Management on missing dose/double dose/uncertainty about dose intake
	Monitoring of anticoagulant activity
Accessibility	Availability of the NOAC
	Price of the NOAC
	Affordability of the NOAC

NOAC, non-vitamin K antagonist oral anticoagulant.

### 2.3 Delphi Survey

The Delphi method is an approach to achieve a convergence of opinion and eventual consensus through multiple iterations of ranking surveys and controlled feedbacks from experts. In this study, the Delphi survey will be conducted according to the Guidance on Conducting and REporting DElphi Studies (CREDES) (Junger et al., 2017). The questionnaire considering candidate components under the six dimensions will be assessed by experts. The survey will be conducted at least two rounds for experts to answer questions and subsequently give justification for their answers. Rounds will continue until the consensus of a pre-defined criterion is achieved. Consensus is defined as greater than 70% agreement on all components. A professional online survey tool (Tencent questionnaire: http://wj.qq.com) will be used to develop the questionnaire. The potential experts will be invited via email, with an explanatory statement of the survey.

#### 2.3.1 Expert Recruitment

A purposive sample of experts who have rich experience in the certain field, i.e., experts in the stroke prevention in AF, as well as experienced pharmacoeconomics will be included in the study. There is no agreement on the sample size in the Delphi survey. It is reported that the Delphi group size depends on group dynamics for arriving at consensus rather than the statistical power (Okoli and Pawlowski, 2004). Typical panels seem to fall into the range of 10–100 experts, consisting of either two or three expert groups (Avella, 2016). Here we take the development of framework for comprehensive evaluation of NOACs in AF as an example. A total of 36 experts from three distinct groups, including clinical pharmacists, cardiologists, and pharmacoeconomists, will be recruited in this study, with 12 experts in each group. More specifically, the participants involved in the Delphi survey need to be established experts in the relevant field, and satisfy the following criteria: 1) having a minimum of 5 years professional experience; 2) clinical pharmacists with expertise in the therapy of cardiovascular drugs or anticoagulants who have obtained their national qualification; 3) cardiologists with expertise in the therapy of AF; 4) pharmacoeconomists who at least being an instructor and skilled at costs and benefits of drugs, and healthcare policies; 5) all included experts need to be interested in this study. Experts will be recruited nationwide in China using a snowball reputation-based sampling procedure. Patients and public will not be involved in the Delphi survey.

#### 2.3.2 First Round of Delphi Survey

Each expert will be asked to rank the importance of every candidate second-level component on a 5-point Likert scale (1 = unimportant, 2 = of little important, 3 = moderately important, 4 = important, 5 = very important) for relevance of inclusion in the framework (Mahajan et al., 2020). A free-text box will be provided for comments or alterations concerning each component. A field for free text will also be provided at the end of the survey for the suggestions of additional components and general comments. Meanwhile, the information on experts’ demographics and professional background will also be collected in the first round. Each survey will take about 30 min to complete and allow participants to review their answers before finial submission. Each survey round will be open for 2 weeks and reminders will be sent 2 days before the deadline.

#### 2.3.3 Second Round of Delphi Survey

All participants will be invited to the second round, including those who do not respond or complete the first round of survey. The results of the first round will be presented anonymously, including the scores and comments of the initial components, as well as any new components being proposed. The experts will be asked to re-score the components and score the new components using the same format as the first round. Components reaching a 70% consensus agreement with a score of four or five will be included in the second level of framework. If the consensus is not achieved by the second round, a third Delphi round will be conducted.

#### 2.3.4 Consensus Meeting

Following the last round, a ranking of component importance will be made to rationalize the number of second-level components in the framework. An online consensus meeting will be organized to discuss the results from the Delphi exercise and finalize the components to be included in the framework. Experts participating all rounds of the survey will be invited to the consensus meeting, and some experts who do not involve in the survey will also be invited. A total of about 20 experts are expected to participate the consensus meeting.

### 2.4 AHP Procedure

Weighting criteria is an important step in the framework development for clinical comprehensive evaluation of drugs (Thokala et al., 2016). Weights represent “trade-off” or “exchange rates” between criteria and bring individual criterion value scores to a common value scale (Thokala et al., 2016). AHP is a powerful tool in multiple criteria decision analysis (MCDA), which can help define weights a hierarchy for criteria in the framework (Saaty, 2008). With AHP, a complex decision can be decomposed into a hierarchy, where the goal is at the top of the hierarchy, the criteria or sub-conditions are at the levels, and the sub-levels of the hierarchy and the potential options are at the bottom of the hierarchy (Boumaiza et al., 2022). A two-level framework will be built in this study, and AHP will be used to prioritize the first-level indicators as well as the second-level components with their weights in the framework. The same Delphi consultation experts will be invited to the AHP procedure.

#### 2.4.1 Pairwise Comparisons

The hierarchy structure will be finalized in the Delphi survey. Pair-wise comparison will be set for each component to another in the same level and will be conducted in the first level and the second level, respectively. For each pair of components, experts will be asked to make comparative judgements on relative importance using a 9-point scale (Table 2), which will translate verbal ratings into a quantitative form (Alharthi et al., 2015). Experts will compare the components in each row with the components in each column. If the two components are of equal importance, the number one will be inserted in the corresponding cell. If the row component is considered more important than the column component, a number between two and nine will be inserted in the corresponding cell (Alharthi et al., 2015; Hooshmand et al., 2015). Conversely, if the column component is considered more important than the row component, the fraction between 1/9 to 1/2 will be inserted in the corresponding cell. Experts will be invited to fill out the questionnaire on pairwise comparisons with the instructions provided, and the data will be collected for the weight determination.

**TABLE 2:** Fundamental 9-point scale for pairwise comparison.

Intensity of Importance	Definition	Description
1	Equal importance	Both items contribute equally
3	Weak importance of one over another	One item is slightly more important than the other
5	Moderate importance	One item is moderately more important than the other
7	Strong importance	One item is strongly more important than the other
9	Extreme importance	One item is extremely more important than the other
2, 4, 6, 8	Intermediate values between two adjacent judgements	Compromised judgement is needed
Reciprocals	If item A is assigned the certain number when compared with item B, the item B is assigned the reciprocal value of the certain number when compared with item A	

#### 2.4.2 Calculation of Weights Coefficient

The relative weight of the components will be calculated by the geometric mean method (Alharthi et al., 2015). The consistency index (CI) and random consistency index (RI) will be used to calculate consistency ratio (CR), which will be adopted to measure the consistency of judgments, with the value <0.1 considered as satisfactory consistency.

### 2.5 Scoring Criteria for Each Second-Level Component

To decide the score of each second-level component, the scoring criteria are needed to be determined. With the evidence of the drug to be evaluated, the score of each component can be obtained according to the established scoring criteria. Delphi survey and AHP will also be used in the determination of scoring criteria.

Each second-level component will be transferred into measurable variables. Moreover, as the framework is designed as evidence-based, the quality of evidence needs to be considered in the scoring criteria, which is important in grading recommendations. Therefore, scoring criteria of components representing therapeutic effects, such as some components under “efficacy” and “safety” dimensions, will involve both score of therapeutic effects and score of evidence level. The weighting ratio of therapeutic effects and quality of evidence will be determined by the AHP procedure. Similarly, the scoring criteria of components representing economic evaluations, such as cost-effectiveness, will involve both results of cost-effectiveness and quality of economic evaluation. The weighting ratio of cost-effectiveness results and quality of economic evaluation will also be determined by the AHP procedure. Experts will be asked to make comparison on relative importance using a 9-point scale (Supplementary Table S2), which will be translated into quantitative weights.

The quality of evidence will be divided into four ratings according to the Grading of Recommendations Assessment, Development, and Evaluation (GRADE) system developed by the GRADE Working Group (Supplementary Table S3), which offers a system for rating quality of evidence and strength of recommendations (Guyatt et al., 2008a; Guyatt et al., 2008b). GRADE is an approach to presenting the quality of the available evidence, the judgements that bear on the quality rating and the effects of alternative management strategies on the outcomes of interest (Guyatt et al., 2011). Irrespective of high or very low quality of evidence, the GRADE approach is applicable (Guyatt et al., 2011). Accordingly, GRADE is considered useful for health technology assessment (Guyatt et al., 2011). In this study, the scoring criteria for evidence levels in GRADE will be explored by the AHP procedure. Pair-wise comparison will be set for each level of evidence to another. Comparative judgements on relative importance between the pair of evidence level will be determined by experts using a 9-point scale (Supplementary Table S4). The score of each level of evidence will be calculated with the methods mentioned above, with the highest level of evidence quality (“high”) obtaining the highest score of 100. Moreover, it is notable that randomized trials concentrate on the efficacy of a drug, while observational studies focus on safety (Kim et al., 2018). Therefore, it is reasonable that different importance of evidence level will be assigned to components belonging to the dimensions of safety and efficacy. To obtain more reasonable and more scientific scoring criteria for evidence levels in this framework, AHP procedure will be conducted for levels of evidence quality in dimensions of safety and efficacy, respectively.

For example, for scoring the component of “reduction of risk for stroke” under the “efficacy” dimension for evaluation of NOACs in AF, experts need to first determine whether the absolute reduction of stroke risk or relative risk reduction compared with warfarin should be used. The score will involve two parts, including therapeutic effects (stroke reduction) and the level of evidence. The proportion will be set as *x*% for therapeutic effects and *y*% for the evidence level, which will be determined in AHP, where *x*% + *y*% = 1. For therapeutic effects, the function is considered linear, and a scoring scale ranging from 0 (the lowest score) to 100 (the highest score) will be used. The range of the possible values of stroke reduction across all anticoagulants will be investigated through the literature review. To accommodate more drugs that will enter the market in the future, the worst utility will be placed at 80% of V_min_ (20% lower than the actual V_min_), while the best utility will be placed at 120% of V_max_ (20% higher than the actual V_max_), in which V_min_ means the worst stroke prevention effect and the V_max_ means the best stroke prevention effect among all anticoagulants. Accordingly, the score of therapeutic effects (stroke reduction) for drug A will be calculated according to the formula: score of therapeutic effects (A) = (V_A_-80%×V_min_)/(120%×V_max_-80%×V_min_)×100. For levels of evidence quality, the level of evidence used to score the therapeutic effects (stroke reduction) will be judged, and the score will be obtained directly according to the scoring criteria of evidence level. Accordingly, the total score of drug A on component of “reduction of risk for stroke” will be calculated as: score (A) = (score of therapeutic effects (A)×*x*%) + (score of quality of evidence (A)×*y*%).

The quality of reporting economic evaluations will be determined by the Consolidated Health Economic Evaluation Reporting Standards (CHEERS) statement, which provides recommendations in the form of a 24-item checklist to optimize reporting of health economic evaluations (Husereau et al., 2013). The quality of economic evaluation will be checked according to the CHEERS statement (Supplementary Table S5), with one item obtaining 4.17 (100/24 = 4.17) scores. The method of scoring cost-effectiveness results is similar to the method of scoring therapeutic effects. The total score of cost-effectiveness will be calculated by aggregating the weighted score of both cost-effectiveness results and the quality of economic evaluation.

The draft of scoring criteria will be formulated according to the above rules. A meeting will also be convened to discuss the preliminary scoring criteria within a committee of experts composing two clinical pharmacists, two cardiologists, and two pharmacoeconomists. One or two rounds of Delphi surveys will be performed to determine the scoring criteria. The recruitment of experts will be the same as the previous rounds. Each expert will be invited to determine whether they agree with these scoring criteria and give recommendations on the specific scoring criteria of each component. Consensus is defined as greater than 70% agreement on each scoring criteria. If the consensus is not achieved by the first round, a second or third Delphi round will be conducted. Finally, a consensus meeting will be held to discuss and finalize the scoring criteria.

### 2.6 Calculation of Comprehensive Score

Scores of each component will be obtained according to the sound evidence of the drug to be evaluated. The comprehensive score will be calculated based on the score of each component and the corresponding weights. First, the weighted score of each second-level component will be obtained by multiplying its score with its weight. The score of each first-level indicator will be obtained by adding all the weighted scores of second-level components under it. Likewise, the weighted score of each first-level indicator will be calculated by multiplying its score with its weight. Finally, the total score will be calculated by aggregating all the weighted scores of first-level indicators. The overall comprehensive score values will be available to the decision makers, which will assist the evidence-based decision making.

### 2.7 Ethics

This study will not involve the health data of individuals, and the ethics approval is not required according to the ethical review of biomedical research involving human subjects in China. Moreover, the study will not collect any sensitive information. Online informed consent will be obtained before completing any questionnaires from all the participants. All the data relevant to this study will be kept on a password-encrypted computer, and only the researchers will have the access to the data.

## 3 Discussion

Clinical comprehensive evaluation of drugs is a complex process, as multiple attributes of drugs including efficacy, safety, cost-effectiveness, etc. need to be considered with different degrees of importance. A scoring framework combining the evidence of different attributes of drugs can help guide the decision making of whether a drug should or should not be listed in NRDL or EDL, by providing a ranking of drugs being evaluated. In this study, we will develop a framework of clinical comprehensive evaluation of drugs using the MCDA, which can provide a structured approach when multiple factors need to be considered systematically and explicitly (Tony et al., 2011). The Delphi survey will be used to achieve a convergence of opinion and eventual consensus on the components need to be evaluated and the scoring criteria of each component. AHP, an MCDA technique, will be used to determine the priority weights of attributes or components in each level (Thokala et al., 2016).

Guided by GCCED, the framework will be built with six attributes as the first-level indicators, including safety, efficacy, costs/cost-effectiveness, novelty, suitability, and accessibility. The second-level indicators will be refined according to the characteristics of the disease and the drug to be evaluated. For example, risk of major bleeding and reduction of risk of stroke are two important indexes for safety and efficacy, respectively, which are specific for anticoagulation therapy in AF patients. Moreover, the weight of each first-level indicators and second-level components can reflect its importance and priority under the upper-level indicator. For example, safety and efficacy were reported to be more important with higher weights than other attributes (Ramli et al., 2013; Yu et al., 2018), as these two indicators are critical factors for drug selection.

Scoring criteria for each component will be carefully designed and determined, which can help differentiate among drugs to be evaluated. Some scoring functions were made flexible at discretion of experts (Yong et al., 2021). Although the reference materials were offered to the experts, the decision-making procedure relied too much on experts’ opinions, which were subjective experience rather than objective evidence (Liu et al., 2019). In this study, we plan to develop the scoring criteria with which scores of each component can be obtained according to the sound evidence of drugs to be evaluated instead of experts’ opinions. In addition, the levels of evidence quality used to score the components will be taken into consideration in the scoring criteria. The levels of evidence rate the quality of scientific evidence, encompassing the estimated magnitude and certainty of benefit in proportion to risk (January et al., 2019), which are important in grading recommendations. Supposing that drug A and drug B present similar therapeutic effects in the certain disease, the evidence of therapeutic effects in drug A is of high quality in GRADE, while the evidence of therapeutic effects in drug B is of moderate quality. The score of the component considering the therapeutic effects will be higher in drug A than drug B, as the level of evidence on therapeutic effects is higher in drug A. Therefore, the levels of evidence quality are crucial in the evaluation of therapeutic effects, which can help differentiate the drugs. Overall, with the scoring criteria to be developed in this study, the objective judgements will be made on each drug, and the results of clinical comprehensive evaluation of drugs can be evidence-based.

The framework that will be developed is for value assessment of drugs. The outcomes of assessment are mainly used to support the pricing decisions on new drugs, or as an aid to make coverage recommendations on the reimbursement status of drugs, as well as assisting rational use of drugs in clinical treatment (Angelis et al., 2018). Therefore, the framework needs to be developed according to different purposes and target users. Different elements need to be evaluated under the perspectives of society, health care or patients (Lakdawalla et al., 2018). For example, assessment frameworks under social perspective need to involve insurance value, severity of disease, and equity, while frameworks under health care perspective do not (Lakdawalla et al., 2018). In this study, the framework will be constructed to support the drug selection into NRDL and EDL for chronic diseases. Nevertheless, the methodology can be a reference to the framework construction for value assessment and clinical comprehensive evaluation of drugs.

## Data Availability

The original contributions presented in the study are included in the article/Supplementary Material, further inquiries can be directed to the corresponding authors.
