# Supplementary material for: Framework Development for Clinical Comprehensive Evaluation of Drugs–a Study Protocol Using the Delphi Method and Analytic Hierarchy Process
Source: Front Pharmacol. 2022 May 19;13:869319. doi: 10.3389/fphar.2022.869319 (PMC9161709; doi:10.3389/fphar.2022.869319)
Supplement: Supplementary file 1 [file DataSheet1.docx]

SUPPLEMENTAL FILE

**Title:** Framework development for clinical comprehensive evaluation of drugs– a study protocol using the Delphi method and Analytic Hierarchy Process

# Table S1. Search strategy to identify studies reporting NOACs in patients with AF

| **Literature databases** | **Search items** | **Items found** |
| --- | --- | --- |
| PUBMED | ("dabigatran"[MeSH Terms] OR "dabigatran"[Title/Abstract] OR "Pradaxa"[Title/Abstract] OR "rivaroxaban"[MeSH Terms] OR "rivaroxaban"[Title/Abstract] OR "Xarelto"[Title/Abstract] OR "apixaban"[Title/Abstract] OR "Eliquis"[Title/Abstract] OR "edoxaban"[Title/Abstract] OR "Savaysa"[Title/Abstract] OR "betrixaban"[Title/Abstract] OR "Bevyxxa"[Title/Abstract] OR "Non-vitamin K antagonist oral anticoagulants"[Title/Abstract] OR "NOACs"[Title/Abstract] OR "direct oral anticoagulants"[Title/Abstract] OR "DOACs"[Title/Abstract] OR "novel oral anticoagulants"[Title/Abstract] OR "new oral anticoagulants"[Title/Abstract] OR "factor Xa inhibitors"[Title/Abstract] OR "factor a inhibitors"[Title/Abstract]) AND ("atrial fibrillation"[MeSH Terms] OR "atrial fibrillation"[Title/Abstract]) | 7034 |
| Web of Science | #1 TS=(dabigatran) OR TS=(Pradaxa) OR TS=(rivaroxaban) OR TS=(Xarelto) OR TS=(apixaban) OR TS=(Eliquis) OR TS=(edoxaban) OR TS=(Savaysa) OR TS=(betrixaban) OR TS=(Bevyxxa) OR TS=(Non-vitamin K antagonist oral anticoagulants) OR TS=(NOACs) OR TS=(direct oral anticoagulants) OR TS=(DOACs) OR TS=(novel oral anticoagulants) OR TS=(new oral anticoagulants) OR TS=(factor Xa inhibitors) OR TS=(factor IIa inhibitors)  #2 TS=(atrial fibrillation)  #1 AND #2 | 14031 |

**Table S2. Pair-wise comparison of "therapeutic effects" with "level of evidence". (Question: Please make comparative judgements on relative importance using a 9-point scale: How important is "therapeutic effects" compared with "level of evidence" in scoring the components representing therapeutic effect of the drug?)**

| **Comparison of therapeutic effects and level of evidence** | | | | | | | | | | | | | | | | | | |
| --- | --- | --- | --- | --- | --- | --- | --- | --- | --- | --- | --- | --- | --- | --- | --- | --- | --- | --- |
|  | **9** | **8** | **7** | **6** | **5** | **4** | **3** | **2** | **1** | **1/2** | **1/3** | **1/4** | **1/5** | **1/6** | **1/7** | **1/8** | **1/9** |  |
| **Therapeutic effects** |  |  |  |  |  |  |  |  |  |  |  |  |  |  |  |  |  | **Level of evidence** |

Note: If you mark "9", you would prefer to consider "therapeutic effects" as "Extreme importance" when compared with "level of evidence". If you think "therapeutic effects" is of “equal importance” with "level of evidence", you should mark “1” in the middle. Similarly, if you think "level of evidence" is of “weak importance” over "therapeutic effects", you should choose “1/3”.

**1** = Equal importance

**3** = Weak importance of one over another

**5** = Moderate importance

**7** = Strong importance

**9** = Extreme importance

**2, 4, 6, 8** = Intermediate values between two adjacent judgements

**Reciprocals:** If item A is assigned the certain number when compared with item B, the item B is assigned the reciprocal value of the certain number when compared with item A.

# Table S3. Significance of the four levels of evidence in GRADE

| **Quality level** | **Definition** |
| --- | --- |
| High | We are very confident that the true effect lies close to that of the estimate of the effect |
| Moderate | We are moderately confident in the effect estimate: The true effect is likely to be close to the estimate of the effect, but there is a possibility that it is substantially different |
| Low | Our confidence in the effect estimate is limited: The true effect may be substantially different from the estimate of the effect |
| Very low | We have very little confidence in the effect estimate: The true effect is likely to be substantially different from the estimate of effect |

**Table S4. Pair-wise comparison of each level of evidence quality to another. (Question: Please make comparative judgements on relative importance using a 9-point scale: How important is each level of evidence quality on the left compared with each level of evidence on the right?)**

| **Comparison of evidence levels** | | | | | | | | | | | | | | | | | | |
| --- | --- | --- | --- | --- | --- | --- | --- | --- | --- | --- | --- | --- | --- | --- | --- | --- | --- | --- |
| **Levels of evidence** | **9** | **8** | **7** | **6** | **5** | **4** | **3** | **2** | **1** | **1/2** | **1/3** | **1/4** | **1/5** | **1/6** | **1/7** | **1/8** | **1/9** | **Levels of evidence** |
| **High** |  |  |  |  |  |  |  |  |  |  |  |  |  |  |  |  |  | **Moderate** |
| **High** |  |  |  |  |  |  |  |  |  |  |  |  |  |  |  |  |  | **Low** |
| **High** |  |  |  |  |  |  |  |  |  |  |  |  |  |  |  |  |  | **Very low** |
| **Moderate** |  |  |  |  |  |  |  |  |  |  |  |  |  |  |  |  |  | **Low** |
| **Moderate** |  |  |  |  |  |  |  |  |  |  |  |  |  |  |  |  |  | **Very low** |
| **Low** |  |  |  |  |  |  |  |  |  |  |  |  |  |  |  |  |  | **Very low** |

**Example:** While evaluating the level of evidence "high" and "moderate" in the first line, if you mark "9", you would prefer to consider "high" as "Extreme importance" when compared with "moderate". If you think "high" is of “equal importance” with "moderate", you should mark “1” in the middle. Similarly, if you think "moderate" is of “weak importance” over "high", you should choose “1/3”.

**1** = Equal importance

**3** = Weak importance of one over another

**5** = Moderate importance

**7** = Strong importance

**9** = Extreme importance

**2, 4, 6, 8** = Intermediate values between two adjacent judgements

**Reciprocals:** If item A is assigned the certain number when compared with item B, the item B is assigned the reciprocal value of the certain number when compared with item A.

**Table S5. The CHEERS checklist**

| **Section/item** | **Item no.** | **Recommendation** |
| --- | --- | --- |
| **Title and abstract** |  |  |
| Title | 1 | Identify the study as an economic evaluation or use more specific terms such as “cost-effectiveness analysis”, and describe the interventions compared. |
| Abstract | 2 | Provide a structured summary of objectives, perspective, setting, methods (including study design and inputs), results (including base case and uncertainty analyses), and conclusions. |
| **Introduction** |  |  |
| Background and objectives | 3 | Provide an explicit statement of the broader context for the study. Present the study question and its relevance for health policy or practice decisions. |
| **Methods** |  |  |
| Target population and subgroups | 4 | Describe characteristics of the base case population and subgroups analysed, including why they were chosen. |
| Setting and location | 5 | State relevant aspects of the system(s) in which the decision(s) need(s) to be made. |
| Study perspective | 6 | Describe the perspective of the study and relate this to the costs being evaluated. |
| Comparators | 7 | Describe the interventions or strategies being compared and state why they were chosen. |
| Time horizon | 8 | State the time horizon(s) over which costs and consequences are being evaluated and say why appropriate. |
| Discount rate | 9 | Report the choice of discount rate(s) used for costs and outcomes and say why appropriate. |
| Choice of health outcomes | 10 | Describe what outcomes were used as the measure(s) of benefit in the evaluation and their relevance for the type of analysis performed. |
| Measurement of effectiveness | 11a | Single study–based estimates: Describe fully the design features of the single effectiveness study and why the single study was a sufficient source of clinical effectiveness data. |
|  | 11b | Synthesis-based estimates: Describe fully the methods used for the identification of included studies and synthesis of clinical effectiveness data. |
| Measurement and valuation of preference-based outcomes | 12 | If applicable, describe the population and methods used to elicit preferences for outcomes. |
| Estimating costs and resources | 13a | Single study–based economic evaluation: Describe approaches used to estimate resource use associated with the alternative interventions. Describe primary or secondary research methods for valuing each resource item in terms of its unit cost. Describe any adjustments made to approximate to opportunity costs |
|  | 13b | Model-based economic evaluation: Describe approaches and data sources used to estimate resource use associated with model health states. Describe primary or secondary research methods for valuing each resource item in terms of its unit cost. Describe any adjustments made to approximate to opportunity costs. |
| Currency, price date and conversion | 14 | Report the dates of the estimated resource quantities and unit costs. Describe methods for adjusting estimated unit costs to the year of reported costs if necessary. Describe methods for converting costs into a common currency base and the exchange rate. |
| Choice of model | 15 | Describe and give reasons for the specific type of decision-analytical model used. Providing a figure to show model structure is strongly recommended. |
| Assumptions | 16 | Describe all structural or other assumptions underpinning the decision-analytical model. |
| Analytical methods | 17 | Describe all analytical methods supporting the evaluation. This could include methods for dealing with skewed, missing, or censored data; extrapolation methods; methods for pooling data; approaches to validate or make adjustments (such as half cycle corrections) to a model; and methods for handling population heterogeneity and uncertainty. |
| **Results** |  |  |
| Study parameters | 18 | Report the values, ranges, references, and, if used, probability distributions for all parameters. Report reasons or sources for distributions used to represent uncertainty where appropriate. Providing a table to show the input values is strongly recommended. |
| Incremental costs and outcomes | 19 | For each intervention, report mean values for the main categories of estimated costs and outcomes of interest, as well as mean differences between the comparator groups. If applicable, report incremental cost-effectiveness ratios. |
| Characterising uncertainty | 20a | Single study–based economic evaluation: Describe the effects of sampling uncertainty for estimated incremental cost, incremental effectiveness, and incremental cost-effectiveness, together with the impact of methodological assumptions (such as discount rate, study perspective) |
|  | 20b | Model-based economic evaluation: Describe the effects on the results of uncertainty for all input parameters, and uncertainty related to the structure of the model and assumptions. |
| Characterising heterogeneity | 21 | If applicable, report differences in costs, outcomes, or cost-effectiveness that can be explained by variations between subgroups of patients with different baseline characteristics or other observed variability in effects that are not reducible by more information. |
| **Discussion** |  |  |
| Study findings, limitations, generalisability, and current knowledge | 22 | Summarise key study findings and describe how they support the conclusions reached. Discuss limitations and the generalisability of the findings and how the findings fit with current knowledge. |
| **Other** |  |  |
| Source of funding | 23 | Describe how the study was funded and the role of the funder in the identification, design, conduct, and reporting of the analysis. Describe other non-monetary sources of support. |
